# Effects of Helical Tube Electrode Structure on Mixed Machining Product Transfer in Micro-Machining Channel during Tube Electrode High-Speed Electrochemical Discharge Machining

**DOI:** 10.3390/mi10100634

**Published:** 2019-09-22

**Authors:** Yan Zhang, Chen Wang, Yu Wang, Lei Ji, Jian Tang, Qin Ni

**Affiliations:** 1 School of Mechanical and Power Engineering, Nanjing Tech University, Nanjing 211800, China; wangchen_fly@njtech.edu.cn (C.W.); jilei.1993@163.com (L.J.); tangjian_1126@163.com (J.T.); niqinpanda@163.com (Q.N.); 2 AECC Sichuan Gas Turbine Research Establishment, Chengdu 610500, China; wangy_cgte@163.com

**Keywords:** electrical discharge machining, electrochemical machining, helical tube electrode, structural optimization, flow field simulation, machining by-products

## Abstract

In tube electrode high-speed electrochemical discharge machining (TSECDM), mixed products are constantly produced in the narrow machining gap by simultaneous discharge erosion and electrochemical dissolution. For the high-efficiency removal of these products from the machining gap, a tool electrode with an optimized helical structure was utilized in TSECDM in this study. Firstly, the concentration distributions of the processed products in the machining gap using tube electrode tools with three typical helical structures were studied through the simulation of solid–liquid coupling; this showed that a trapezoidal helical structure benefited the reduced accumulation of products in grooves and the effective removal of products from the machining gap. Secondly, the main geometric parameters of the trapezoidal helical structure, including the helical groove depth, pitch, and tooth angle, were optimized by gap flow-field simulation to enhance the removal effect on processed products. Finally, it was verified that the trapezoidal helical electrode showed a definitive and significant advantage over the ordinary cylindrical electrode in effectively removing processed products from the machining gap by the comparison of flow-field simulations and actual machining experiments.

## 1. Introduction

To improve heat dissipation and working reliability, gas film-cooling holes are widely used in aero-engine turbine blades [1,2]. Tube-electrode high-speed electrochemical discharge machining (TSECDM), a hybrid manufacturing method of electrical discharge machining (EDM) and electrochemical machining (ECM) that exploits the advantages of these two technologies to achieve high machining efficiency [3], machining accuracy [4], and surface quality [5], is potentially applicable for the fabrication of film-cooling holes. However, complex mixed machining by-products, including both molten metal particles and electrochemically dissolved materials, are continuously produced in the narrow machining gap [6,7]. This greatly increases the risk of excessive product accumulation and the deterioration of the flow field environment, thus seriously reducing the processing quality of the holes [8,9].

To improve the flushing conditions, the tool electrode is always considered a vital component, especially in the hybrid TSECDM process. Liu et al. utilized a tool electrode with a helical structure to conduct short-pulse electrochemical drilling, successfully realizing the fabrication of taper-free micro-holes [10]. Wang et al. used a wedge tube electrode for electrochemical drilling to improve the uniformity of the flow-field distribution in the machining gap, obtaining high-quality inclined holes with large inclination angles [11]. Yang et al. developed a method for electrochemical discharge drilling using a spherical tool electrode [12]. The improved machining effect is attributed to the spherical structure accelerating the flow of the working fluid to the end of the electrode, thus allowing for the rapid formation of the gas film. Hung et al. proposed a machining method of micro-EDM that matched the rotational helical tool electrode with the ultrasonic disturbance of the flow field, obtaining micro-hole structures with high surface quality and machining precision with the optimum processing parameters [13]. Nastasi and Koshy studied the effect of different tool electrode geometries on blind-hole machining by electrical discharge drilling. Through comparative experiments, they concluded that the electrode with a radial slot had a better material removal rate than a cylindrical electrode [14]. Plaza et al. investigated the effect of the geometric parameters of helical electrodes on micro-EDM drilling performance, experimentally showing the effective removal of the debris from the machining gap as the machining depth increased [15]. Hung et al. proposed a helical solid electrode with an insulation layer for electrochemical micro-drilling. Secondary electrochemical corrosion was effectively avoided, while the removal of machining products was enhanced [16]. Fang et al. introduced a helical cylindrical electrode to wire electrochemical micromachining in order to facilitate electrolyte refreshment in the machining gap, achieving microstructure fabrication with good uniformity [17].

TSECDM, as a hybrid of electrical discharge and electrochemical machining, yields processing products that include molten metal particles from electrical discharge and flocculent hydroxides from electrochemical dissolution. Therefore, the removal of these processing products from the narrow machining gap is difficult. In this study, a tool electrode with a specially optimized helical structure was utilized in TSECDM to overcome this challenge. Firstly, the concentration distributions of the processed products in the machining gap using the tube electrode tools with three typical helical structures were studied to identify the best helical structure for product removal through simulated solid–liquid coupling. Secondly, the main geometric parameters of trapezoidal helical structures, including the helical groove depth, pitch, and tooth angle, were optimized to improve the effect of removing processed products by gap flow-field simulation. Finally, it was verified that the trapezoidal helical electrode showed a definite and significant advantage over the ordinary cylindrical electrode in effectively removing processed products from narrow machining gaps by the comparison of flow-field simulations with actual machining experiments.

## 2. Materials and Methods

### 2.1. Improvement of TSECDM of Holes by Using Helical Tube Electrode

**Figure 1:**
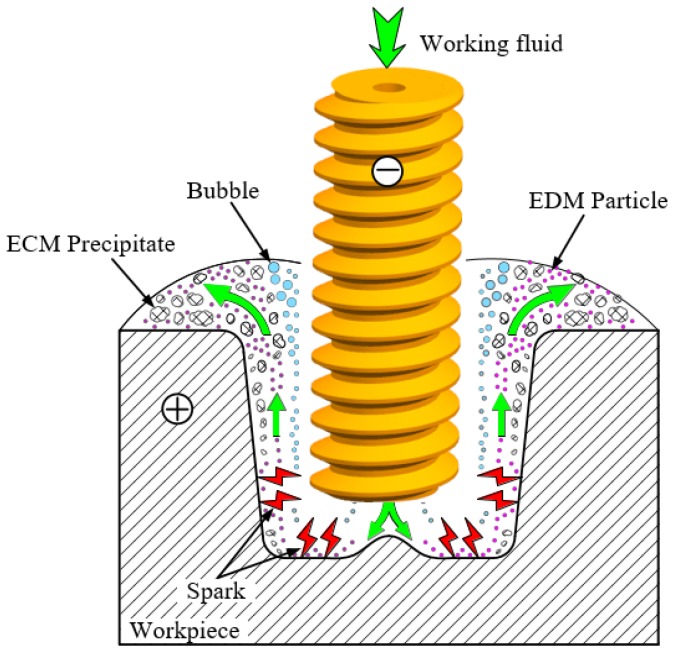
Principle of improvement of tube electrode high-speed electrochemical discharge machining (TSECDM) of holes by using helical tube electrode.

In TSECDM, the mixed machining products are difficult to effectively remove from the narrow machining gap, which seriously affects the machining accuracy and surface quality of holes. To solve this problem, a helical tube electrode was proposed for use in this hybrid machining process for high-quality holes. Unlike ordinary tube electrodes, the tool electrode utilized in TSECDM is a tube with an optimized helical structure. During the process of hole fabrication, the helical electrode is rotated at high speed to stir the working fluid inside the machining gap, enhance the flushing effect, and promote the rapid removal of mixed processing products, as shown in Figure 1. Meanwhile, the flow field near the helical structure sucks some of the machining products into the helical groove. These drain along the surface of the helical groove under the extrusion action of the helix, thereby greatly reducing the agglomeration of the products in the narrow machining gap. Enhancing the processing stability effectively improves the hole machining performance.

### 2.2. Experimental Setup

#### 2.2.1. Helical Structure Design of Tube Tool Electrode

**Figure 2:**
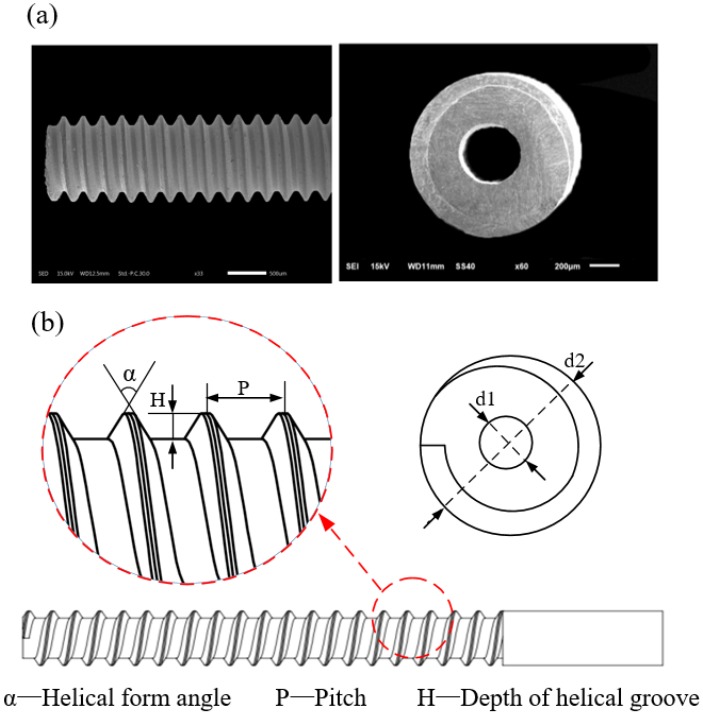
(**a**) Physical images of the trapezoidal helical tube electrode and (**b**) geometric parameters of the helices.

A helical structure is used for the tool electrode in TSECDM. To promote the removal efficiency of mixed machining products produced by hybrid machining and obtain high-quality holes, the helical structure must be specially designed. Helical structures of tube tool electrodes are designed using a constant groove volume. Different helical structures such as rectangular, triangular, and trapezoidal helices are used as base geometries. For the trapezoidal helical structure on the surface of the tube electrode, an optimization analysis of its major geometric parameters, such as the thread form angle, helical pitch, and depth of helical groove, was performed. The trapezoidal helical tube electrode is shown in Figure 2a, and the geometric parameters of the trapezoidal helical structure are illustrated as shown in Figure 2b.

#### 2.2.2. Experimental System

**Figure 3:**
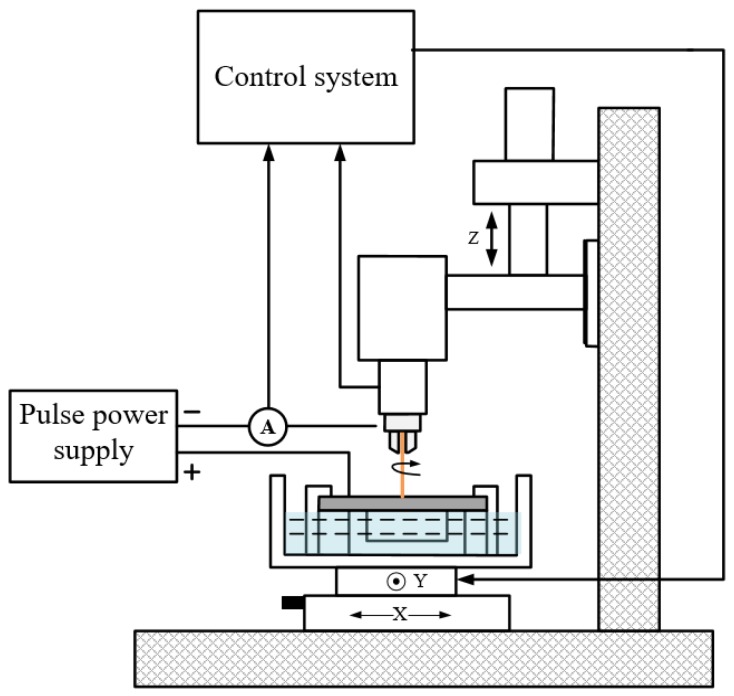
Schematic of experimental system.

Figure 3 shows the schematic of the experimental system, mainly comprising a power supply system, a control system, and a machining system. A high-frequency pulsed power supply was used to obtain good-quality holes. The control system manipulates the high-pressure pump to supply the working fluid and controls the high-precision movement of the spindle and machine table. The processing system includes the workpiece, tube tool electrode, fixtures, and so on.

#### 2.2.3. Simulation and Experimental Parameters Setting

To illustrate the effects of different helically structured tube electrodes on the removal of machining products from the narrow machining gap, flow-field simulations of TSECDM with helical electrodes were performed. During the fabrication process, both molten metal particles with a diameter of 5–20 μm and electrochemical dissolution products with a diameter of 20–50 μm are generated in the machining gap. Therefore, to match the actual situation as closely as possible, the particle size of the processed products was set to 10 different values, varying randomly within the range 5–50 μm in the simulation. Other simulation parameters are shown in Table 1. The experimental parameters are listed in Table 2.

#### 2.2.4. Mathematical Model of Flow Field in Machining Gap

In order to simplify the simulation of the gap flow field in the process of micro-hole fabrication, the following assumptions were made: The fluid in the gap flow field is an ideal continuous medium, and after flow field stabilization, the physical quantities are only related to the coordinates.

The flow of working fluid in the machining gap follows the conservation of mass. Because the density of an incompressible fluid is constant, the continuity equation can be described as follows:

$$\frac{\partial u_{x}}{\partial x}+\frac{\partial u_{y}}{\partial y}+\frac{\partial u_{z}}{\partial z}=0.$$

In addition to satisfying the continuity equation, the working fluid in the machining gap also satisfies momentum conservation and can be described as follows [18]:

$$\begin{array}{l} \frac{\partial (\rho u_{x})}{\partial t}+\nabla (\rho u_{x}\vec{u})=-\frac{\partial p}{\partial x}+\frac{\partial \tau_{xx}}{\partial x}+\frac{\partial \tau_{yx}}{\partial y}+\frac{\partial \tau_{zx}}{\partial z}+\rho f_{x}\\ \frac{\partial (\rho u_{y})}{\partial t}+\nabla (\rho u_{y}\vec{u})=-\frac{\partial p}{\partial y}+\frac{\partial \tau_{xy}}{\partial x}+\frac{\partial \tau_{yy}}{\partial y}+\frac{\partial \tau_{zy}}{\partial z}+\rho f_{y}\\ \frac{\partial (\rho u_{z})}{\partial t}+\nabla (\rho u_{z}\vec{u})=-\frac{\partial p}{\partial z}+\frac{\partial \tau_{xz}}{\partial x}+\frac{\partial \tau_{yz}}{\partial y}+\frac{\partial \tau_{zz}}{\partial z}+\rho f_{z} \end{array}$$

With energy transfer ignored, the continuity equation and momentum conservation equation constitute the mathematical model of the gap flow field.

The processed products are driven by the flow field in the inter-electrode gap to acquire velocity and move. They are mainly subject to fluid force and gravity in the flow field. Newton’s second law can be applied to the particles per unit mass to obtain their dynamic equation [19]:

$$\frac{d{\vec{u}}_{p}}{dt}=\vec{F}+\vec{g}(1-\frac{\rho }{\rho_{p}})+\frac{18C_{D}R_{e}\mu }{24\rho_{p}d_{p}^{2}}(\vec{u}-{\vec{u}}_{p})$$

## 3. Results and Discussion

### 3.1. Concentration Distributions of Hybrid Products in the Small Machining Gap Using Tube Electrodes with Different Helical Structures

In TSECDM, significant molten metal particles and electrochemically-dissolved products are continuously produced in a narrow machining gap. To further illustrate the role of the helical structure on promoting the removal of mixed products in such a machining gap, simulations using the method of solid–liquid coupling were conducted to determine the concentration distributions of products in the machining gap.

**Figure 4:**
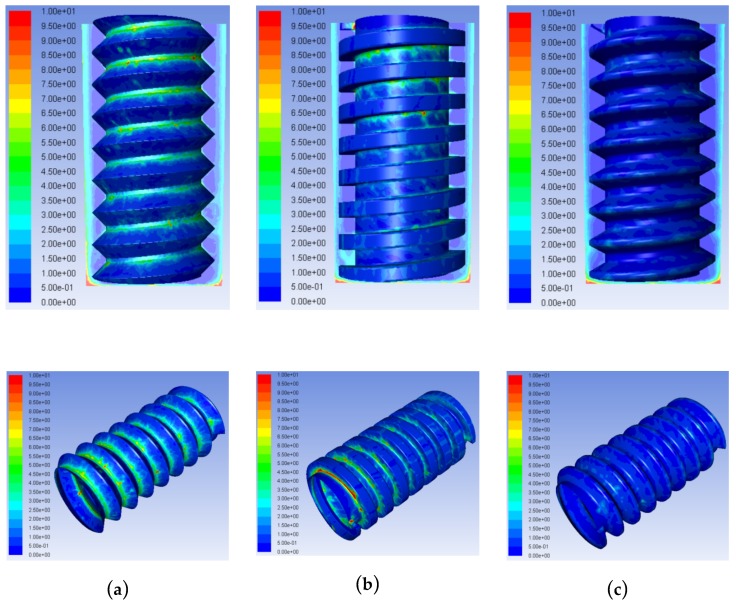
Concentration contours of processed products in the machining gap using tool tube electrodes with different helical shapes: (**a**) Triangle; (**b**) rectangle; and (**c**) trapezoid.

Figure 4 shows the concentration distributions of processed products in the machining gap of TSECDM using helical tube electrodes with three different shapes. The effects of different helical structures on the product distributions in the machining gaps differed significantly. The amount of processing products using the triangular electrode was the largest, as shown in Figure 4a; the concentration in the side machining gap was 6.5 kg/m^3^, and the products almost filled each thread groove. For the triangular helical electrode, the energy loss of the gap fluid caused by the obstruction of the helical structure was the largest under the same flushing pressure; this led to the fastest velocity decline of the working fluid mixed with processing products near the groove. In addition, the axial component force of the extruded fluid moving towards the entrance of the hole was the lowest during electrode rotation. For the rectangular helical electrode, high-velocity eddies in the direction opposite to the flushing working fluid in the side machining gap impeded the movement of the processed products in the helical groove. Meanwhile, the larger tooth form angle of the rectangular helix was not conducive to product removal. The concentration of products in the side machining gap was 3.5 kg/m^3^, as shown in Figure 4b, and more processing products were concentrated in the helical grooves near the hole entrance. For the trapezoidal helical electrode, the special helical shape structure yielded a relatively moderate disturbance effect of the electrode on the working fluid in the machining gap. The pressure difference between the internal and external fluid in the threaded groove was similarly moderate, allowing for the extremely efficient removal of the processed products.

**Figure 5:**
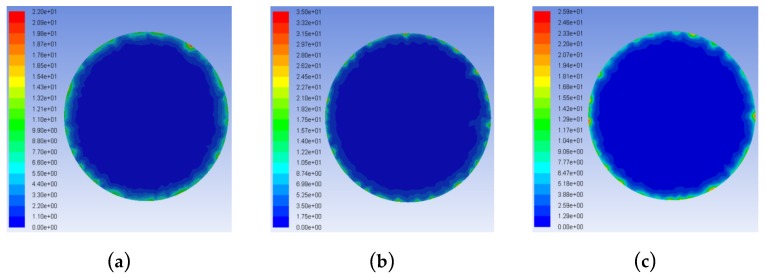
Concentration distributions of the processed products in the frontal machining gap: (**a**) Trapezoid; (**b**) rectangle; and (**c**) triangle.

Figure 5 shows the concentration distributions of the processed products in the frontal machining gap. The product concentration distributions in the processing gap were relatively high with the triangular and rectangular helical electrodes, with the maximum product concentrations reaching 25 and 50 kg/m^3^, respectively. When the trapezoidal helical electrode was used, the maximum product concentration in the machining gap was only 10 kg/m^3^, which was 15 kg/m^3^ lower than that with the triangle helix and 40 kg/m^3^ lower than that with the rectangular helix. This illustrates that the trapezoidal helical structure was the most helpful in avoiding the accumulation of processed products at the electrode edges and corners. This was consistent with the results of product distributions shown in Figure 4. The reduced agglomeration of complex mixed products in the narrow machining gap effectively reduced abnormal discharge, which is conducive to improving the machining accuracy and quality of the hole.

**Figure 6:**
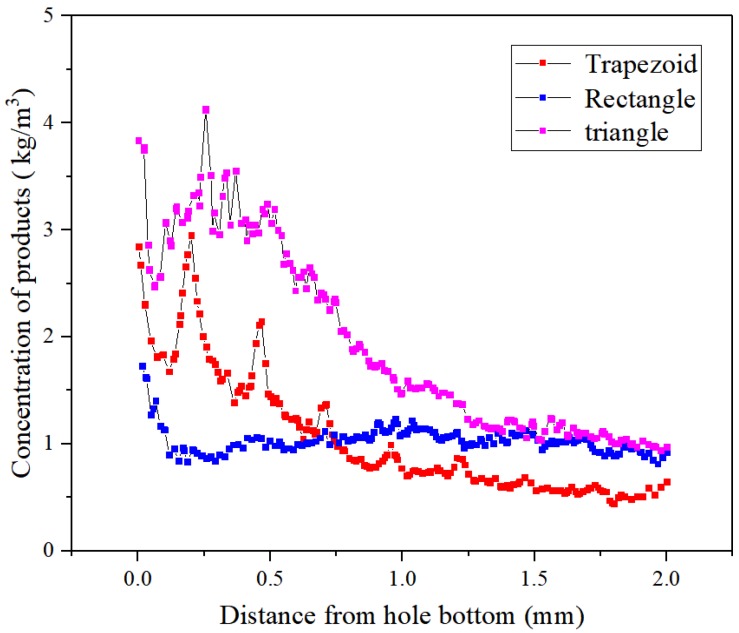
Concentration distributions of the processed products in the side machining gaps using different helical electrodes.

Figure 6 reflects the variation of the concentration distributions of the processed products in the side machining gap using different helical electrodes. With the three geometries, the concentration curves all showed an overall decrease with the increasing distance from the bottom of the hole. Nevertheless, the product concentrations showed significant fluctuations when using the triangular and trapezoidal helical electrodes within 0–0.5 mm from the bottom of the blind hole. This indicates that, compared with the straight rectangular structure, the triangular and trapezoidal helical electrodes provided stronger disturbances to the flow field in the machining gap because of their special thread form angle structures. However, the processed products using triangular and rectangular helical electrodes were more distributed in the lateral machining gap >1 mm from the bottom of the hole, with similar concentration values close to 1.2 kg/m^3^. The concentration value in this regime with the trapezoidal helical electrode was approximately 50% of those with the other two geometries. These results are consistent with those shown in Figure 4, further indicating that the trapezoidal helical electrode benefits from the removal of gap products.

**Figure 7:**
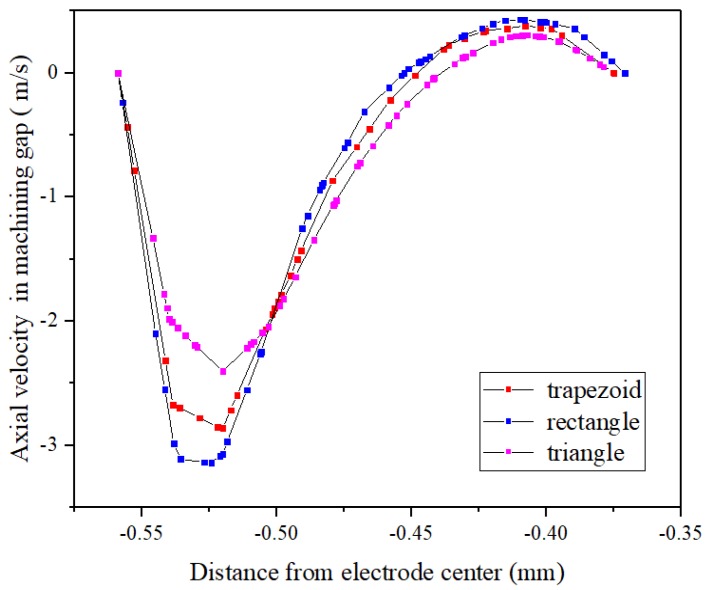
Axial velocity distributions in lateral machining gap with different helical electrodes.

Figure 7 shows the velocity distributions of the working fluids in the lateral machining gap using different helical electrodes. The change in velocity direction was caused by eddy movement from the helical structure stirring the flow field. The flow velocity of the rectangular helical structure showed the largest variation both inside and outside the helical groove, indicating that the energy loss of fluid movement was the largest, which was consistent with the results in Figure 6. Simultaneously, Figure 7 shows that the velocity with a triangular helical electrode in the machining gap was the lowest among the three electrode structures, further supporting the results of excessive product accumulation in the helical grooves and machining gap. Through this comparative analysis, the trapezoid was shown as the optimal helical structure because the flow velocity distribution both inside and outside the helical grooves in the narrow machining gap was the most moderate.

Hence, according to the results in Figure 4, Figure 5, Figure 6 and Figure 7, it can be concluded that, compared to the other two helical structures, the tube electrode with a trapezoidal helical structure was the most conducive to avoiding excessive product accumulation in the helical groove, thus allowing for the effective product removal from the narrow machining gap.

### 3.2. Optimization of Main Geometric Parameters for Trapezoidal Helical Tube Electrode

#### 3.2.1. Effect of Helical Pitch on Product Concentration Distribution in the Machining Gap

In order to further promote the removal of machining products from the narrow machining gap and reduce product accumulation in the helical grooves, the main geometric parameters of the trapezoidal helical structure were optimized. A flow field simulation of solid–liquid coupling was used for the optimization of the depth of helical grooves, the helical pitch, and the thread form angle to improve the machining accuracy and quality of holes machined with a trapezoidal tube electrode.

**Figure 8:**
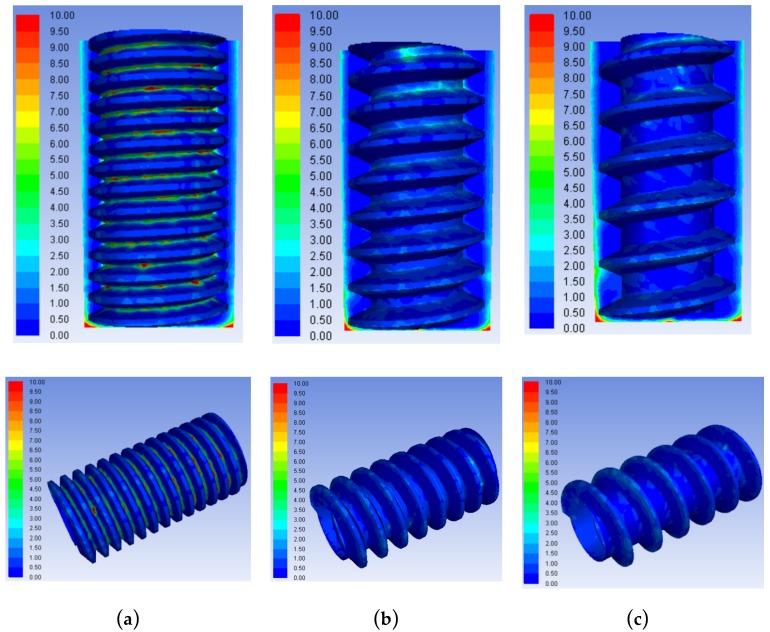
Concentration contours of processed products in the machining gaps using trapezoid helical electrodes with different pitches: (**a**) 0.15 mm; (**b**) 0.25 mm; and (**c**) 0.35 mm.

**Figure 9:**
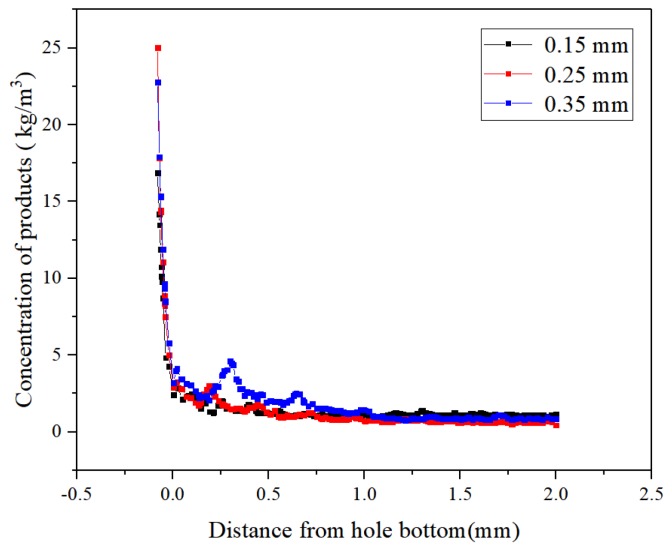
Concentration distributions of the processed products in the axial direction of the hole in the side machining gaps using trapezoidal helical electrodes with different pitches.

Figure 8 and Figure 9, respectively, show the concentration distribution contours and the product concentration curves in the machining gaps using tube electrodes with different helical pitches. At the pitch of 0.35 mm, the small number of helixes reduced the disturbance effect of the helical structure on the flow field in the machining gap, which did not assist the efficient removal of products from the machining gap. In addition to product accumulation in the helical groove and product adhesion on the top surfaces of the helical teeth, an abnormal increase in product concentration also occurred in the machining gap, as shown in Figure 9. The variation of the product concentration with the distance from the hole bottom in the machining gap in Figure 9 also demonstrates that the 0.35 mm pitch did not facilitate the dispersive distribution of processed products in the machining gap. The concentration of local products reached 5 kg/m^3^, twice that with the other two pitches. Excessive product accumulation in the machining gap seriously deteriorated the machining accuracy and quality of holes. Figure 8 shows that the helical pitch of 0.15 mm yielded an excessive number of helical teeth. These caused an excessively strong disturbance of the working fluid in the machining gap, leading to an excessive fluid pressure difference inside and outside the helical groove with significant product accumulation within the grooves. Figure 9 shows that the concentration of processed products in the machining gap at the pitch of 0.25 mm was similar to that at the 0.15 mm pitch. With a 0.25 mm pitch, the processed products only partially accumulated in the helical grooves near the hole entrance, but the concentration and quantity of the processed products were significantly lower than that with a 0.15 mm pitch.

Therefore, it can be concluded from the results in Figure 8 and Figure 9 that, for the trapezoidal helical electrode, a large or small pitch was not conducive to the effective removal of processing products. In order to obtain holes with higher machining accuracy and surface quality, the optimal pitch of the trapezoidal helical electrode is 0.25 mm.

#### 3.2.2. Effect of Thread Form Angle on Product Concentration Distribution in Machining Gap

**Figure 10:**
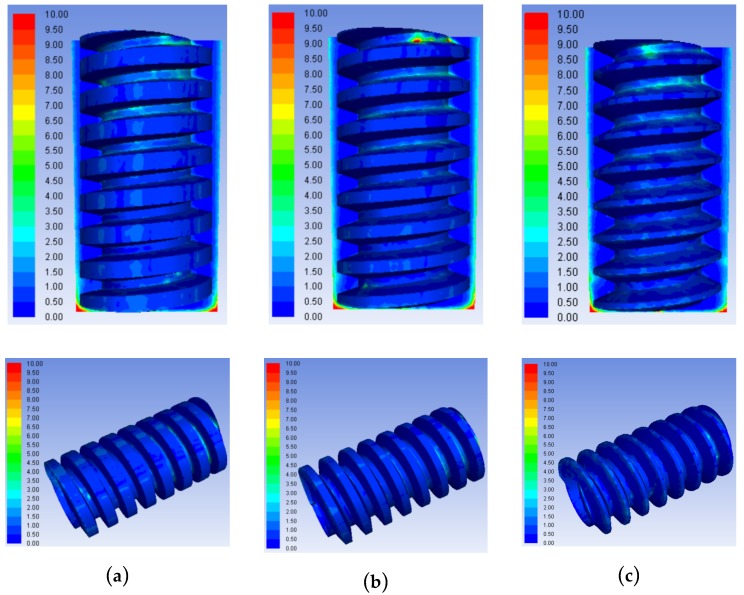
Concentration distributions of processing products in machining gaps and helical grooves using tube electrodes with different thread form angles: (**a**) 30°; (**b**) 45°; and (**c**) 60°.

**Figure 11:**
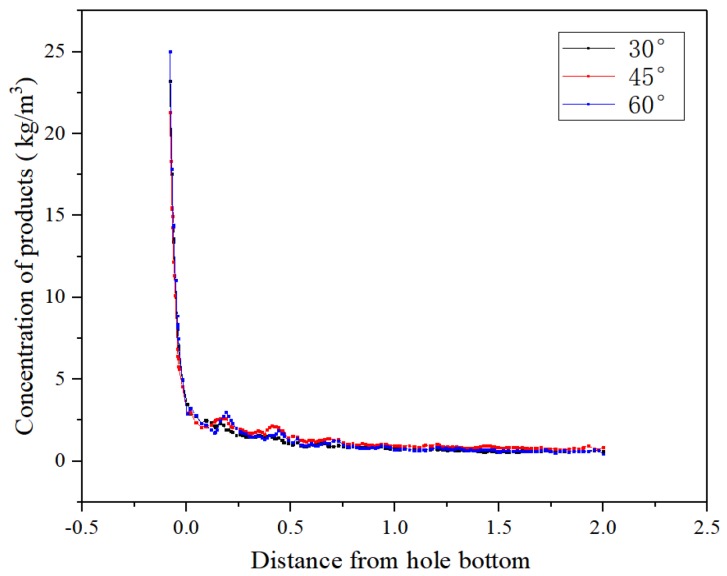
Concentration variations of processed products in the axial direction of the hole in the machining gap using different thread form angles.

Figure 10 presents the concentration distributions of processing products using trapezoidal helical thread electrodes with different tooth angles. At the tooth profile angle of 30°, because of the large contact area between the top of the thread and the working fluid, the high-viscosity mixed product easily adhered to the top surfaces of the helices. Significant product accumulation occurred in the grooves near the hole entrance. The product adhesion on the top surfaces of the helices was reduced at the tooth form angle of 45°, but the highest concentration of product was observed in the helical grooves of this electrode. However, at the thread form angle of 60°, the larger inclined angle of the tread flank yielded some product aggregation in the grooves and the partial adhesion of product to the top surfaces of the helices. In addition, according to the results for the concentration variation of processed products in the axial direction of the hole in the machining gap shown in Figure 11, no significant difference was observed in the product concentration distributions in the processing gap using trapezoidal electrodes with these three thread form angles.

Therefore, the comparative analysis of Figure 10 and Figure 11 shows that the best tooth angle for the trapezoidal helical structure is 60° to obtain the best product removal effect.

#### 3.2.3. Effect of Depth of Helical Groove on Flow Field in Machining Gap

**Figure 12:**
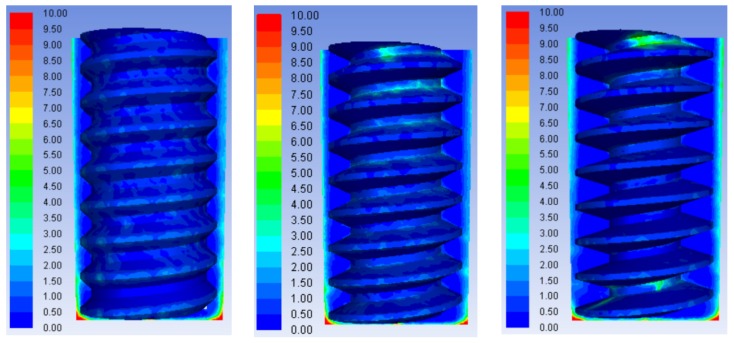

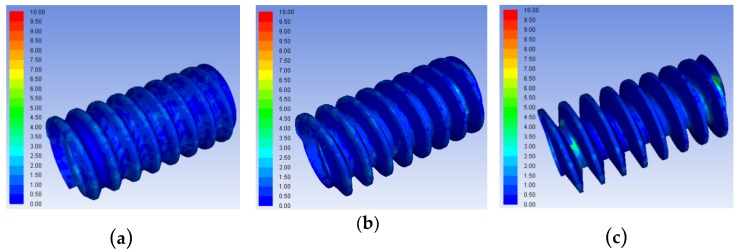
Concentration contours of processing products in machining gap using trapezoidal helical electrodes with different helical groove depths: (**a**) 0.08 mm; (**b**) 0.16 mm; and (**c**) 0.24 mm.

**Figure 13:**
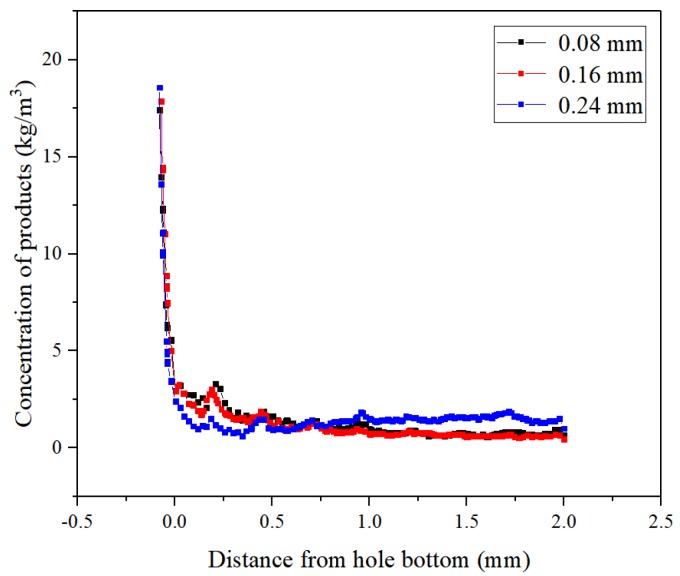
Concentration variation of products in the axial direction of the hole in machining gap using trapezoidal helices with different helical groove depths.

The contours of the product concentration distributions and their axial concentration variations in the machining gap with different helical groove depths are shown in Figure 12 and Figure 13, respectively. As shown in Figure 12, with the helical groove depth of 0.24 mm, the excessive depth caused excessive product accumulation in the helical groove near the hole entrance, with product concentration reaching 5.5–6 kg/m^3^. Figure 13 also clearly shows that the 0.24 mm deep helical groove yielded higher product concentration in the machining gap than those with the other two groove depths. This illustrates that excessive groove depth not only caused product accumulation but also impeded product removal from the narrow lateral machining gap. With a 0.08 mm deep groove, a slight product distribution with a concentration of 1–1.5 kg/m^3^ was observed at the top of the helical teeth, while the product distribution in the grooves was low. The product concentration distribution in the processing gap was similar to that with the electrode having a 0.16 mm deep groove. With the helical groove depth of 0.08 mm, the helical structure had a moderate disturbance effect on the flow field in the machining gap, and the fluid pressure difference inside and outside the helical groove was appropriate, thus yielding the best product removal effect. Therefore, the optimal depth of the trapezoidal helical groove is 0.08 mm.

Hence, considering the effects of groove depth, pitch, and thread form angle on the removal effects of processing products, the final optimized geometric parameters for the trapezoidal helical structure are a groove depth of 0.08 mm, a pitch of 0.25 mm, and a thread form angle of 60°.

### 3.3. Comparison of Flow Field Simulations and Experiments Using Trapezoidal Helical and Cylindrical Tube Electrodes

**Figure 14:**
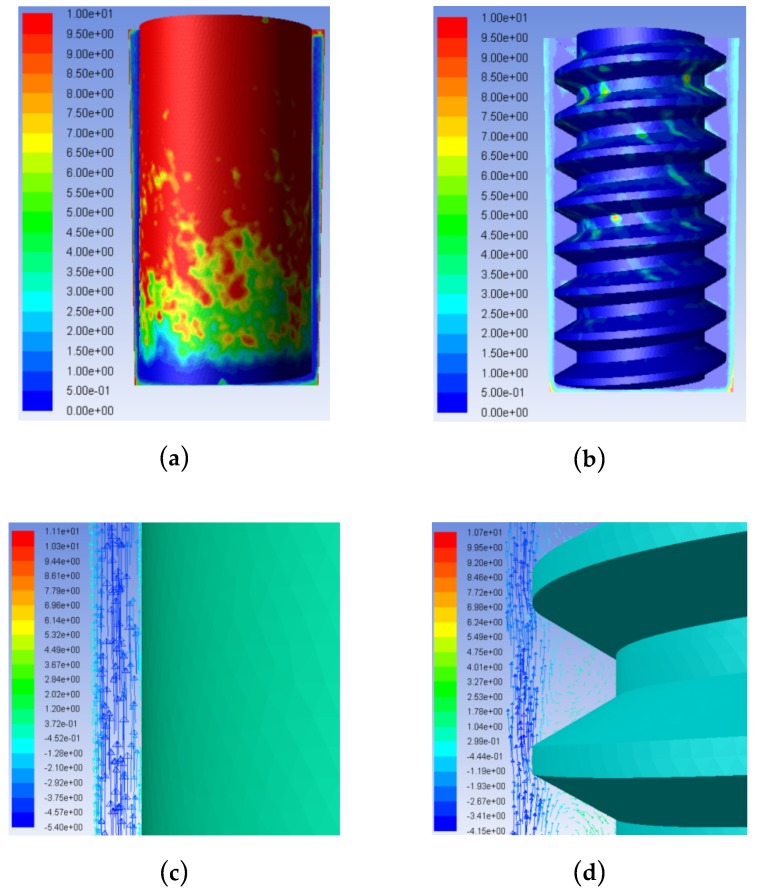
Comparison of simulation results with different tool tube electrodes: Product concentration contours with (**a**) trapezoidal helical electrode and (**b**) ordinary cylindrical electrode. Vectors of velocity in machining gap with (**c**) trapezoidal helical electrode and (**d**) ordinary cylindrical electrode.

By comparing the results in Figure 14a,b, it can be seen that the influence of different tube electrode structures on the product concentration distribution in the machining gap differed significantly. With a cylindrical tube electrode, the product concentration increased along the surface of the cylindrical tube electrode from the bottom to the entrance of the hole, with the maximum concentration of 10 kg/m^3^ observed over nearly 70% of the electrode surface area. This was mainly because the smooth external surface of the ordinary cylindrical tube electrode cannot change the state of the gap flow field. The working fluid in the machining gap was always laminar in flow, as shown by the velocity vectors in Figure 14c, and the mixed processing products could only be removed through gap flushing. The processed products were generated quickly in a short time, and the saturated mixed products soon reached the limit capacity of flushing removal, allowing for significant product accumulation. With the trapezoidal helical electrode, product distribution in the side processing gap reached a concentration of only 4–5 kg/m^3^. In the helical grooves, product concentration only reached 1–2 kg/m^3^ in a few locations. This was because the high-speed rotation of the helical structure strengthened the disturbance of the gap flow field, causing the working fluid in the machining gap to change from laminar to turbulent flow, which promoted product removal. Simultaneously, some products were squeezed into the grooves by eddy currents formed near the grooves. Finally, machining products were removed to the orifice along the helical line through the extrusion of the helical structure. Hence, through the comparison and analysis of the above simulation results, it can be concluded that the helical tube electrode is significantly better than the ordinary cylindrical tube electrode in terms of product removal capacity.

**Figure 15:**
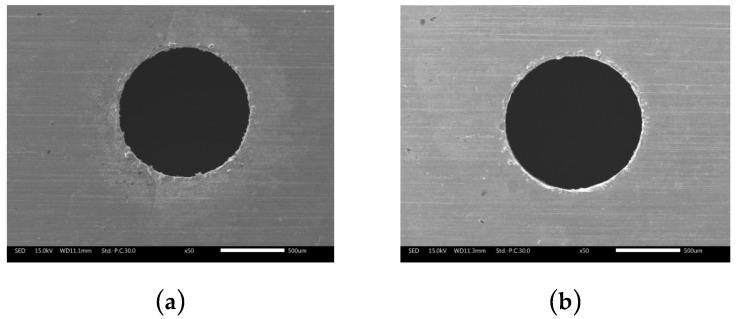
Orifice morphologies of holes processed with different tube electrodes: (**a**) Cylindrical tube electrode; (**b**) helical tube electrode.

**Figure 16:**
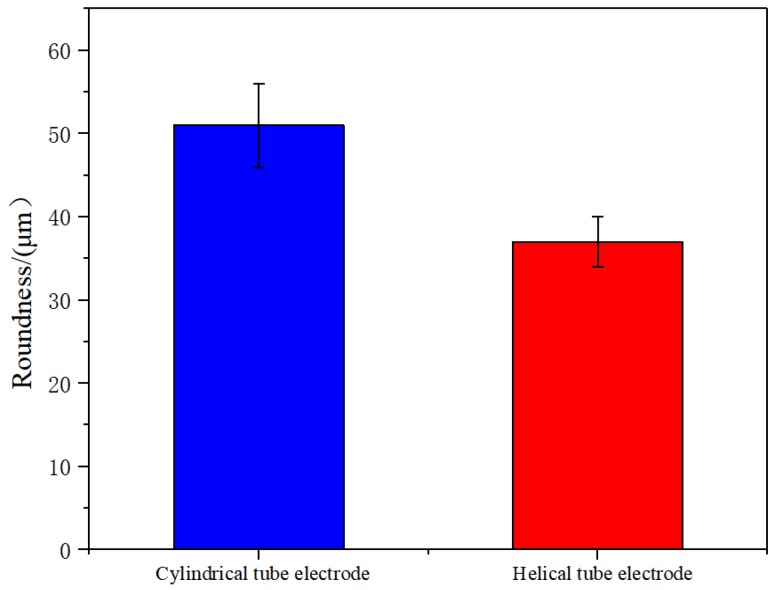
The comparison of roundness error by different tube electrodes.

Figure 15 shows the orifice morphologies of holes processed by TSECDM with different tube electrodes. By comparing the morphologies of the holes in Figure 15a,b, it can be seen that the diameter of the hole machined by the helical electrode was enlarged by 8.2%. Moreover, the shape of the orifice was more regular, with the roundness error reduced from 51 to 37 μm, as shown in Figure 16. This can be attributed to the fact that the helical tube electrode promoted the removal efficiency of processed products, which not only improved the material removal rate but also enhanced the uniformity of electrochemical corrosion at the orifice. The smooth surface of the cylindrical tube electrode had no positive effect on products removal, as reflected in Figure 14a, so the machining accuracy was naturally not as good as that of the helical tube electrode.

**Figure 17:**
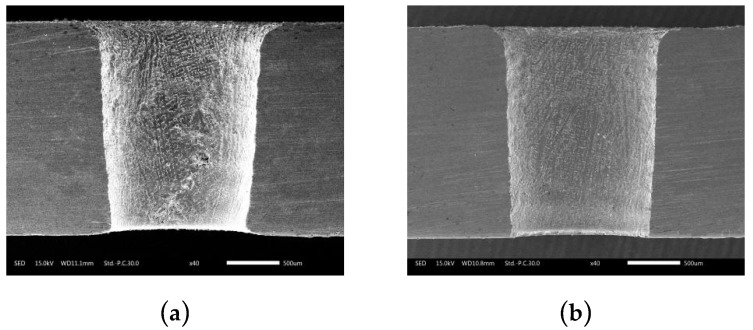
Sectional views of holes machined by different tube electrodes: (**a**) Cylindrical tube electrode; (**b**) helical tube electrode.

The sectional views of holes machined by different tube electrode are shown in Figure 17. In terms of taper, as observed in Figure 17a,b, the hole processed by the trapezoidal helical electrode obviously showed a lower taper than that by the ordinary cylindrical electrode. This was mainly because the rotating helical tube electrode effectively accelerated the removal of products in the small machining gap and prevented the local excessive accumulation of products near the frontal area of tool electrode, as shown in Figure 14. The uniformity of particle concentration in the whole lateral machining gap from the entrance to the exit was well improved; thus, the taper resulted from the difference in material removal volume between entrance and exit of the hole could be effectively reduced.

**Figure 18:**
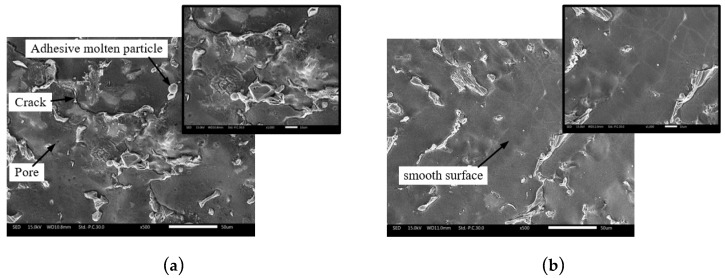
The morphologies of hole wall processed by the different electrodes: (**a**) Cylindrical tube electrode; (**b**) helical tube electrode.

The morphologies of hole wall processed by the cylindrical tube electrode and the helical tube electrode are shown in Figure 18a,b, respectively. It can be seen from Figure 18a that the hole surface machined by cylinder tube electrode was relatively poor and included various defects like adhesive molten particles, micro cracks, and so on. The generation of rough defect layer can be interpreted from two aspects. On one hand, due to the poor removal of machining products, some molten spark products easily adhered to the machined surface. On the other hand, excessive products accumulated in the machining gap could seriously destroy the hybrid machining stability and lead to abnormal discharge; thus, the poor machining surface quality could be obtained. However, the helical tube electrode could effectively disturb the flow field in the machining gap, as illuminated by simulation results in Figure 14d. The product elimination in the machining gap and the rapid update of fresh working fluid could significantly improve the processing performances. Therefore, it can be observed in Figure 18b that the hole wall surface processed by helical tube electrode was relatively smooth.

Therefore, combining the comparison results of the above three aspects, it can be concluded that the helical electrode contributes to products removal so as to gain a hole with better shape accuracy and surface processing quality.

## 4. Conclusions

In this study, focusing on the effective removal of complex mixed products from narrow machining gaps, a TSECDM tool electrode with a helical structure was proposed and demonstrated. The main conclusions are summarized as follows:

1. Flow-field simulations of tube electrodes with different helical structures show that, compared to triangular and rectangular helical structures, less product aggregation in the helical grooves occurs using a trapezoidal helical electrode, which is most beneficial for product removal.
2. Through the simulation and optimization of the main geometric parameters of the trapezoidal helical structure, the optimal groove depth is 0.08 mm, the pitch is 0.25 mm, and the tooth angle is 60°.
3. The flow field simulation of the machining gap between the trapezoidal helical electrode and the cylindrical electrode shows that the trapezoidal helix is more beneficial for product removal. The simulation results are supported by experimental data on machined holes.

## Figures and Tables

**Table 1:** Parameters of flow-field simulation for different helically structured tube electrodes.

Parameter	Value
External diameter of helical tube electrode (μm)	1000
Internal diameter of helical tube electrode (μm)	300
Flushing pressure (m/s)	8
Rotation of helical tube electrode (r/min)	600
Particle diameter (μm)	5–50

**Table 2:** Parameters of hole machining for different tube electrodes.

Parameter	Value
Thickness of workpiece (mm)	2
Working fluid concentration of NaNO_3_ (g/L)	4.5
Flushing pressure (MPa)	3.5
Rotation of helical tube electrode (r/min)	300
Pulse duration (μs)	12
Pulse interval (μs)	12
Peak current (A)	14

## Nomenclature

*u_x_*, *u_y_*, *u_z_*	velocity components in the *X*, *Y*, *Z* directions (m/s)
*t*	fluid flow time (s)
*ρ*	fluid density (kg/m^3^)
$\tau_{xx}$, $\tau_{xy}$, $\tau_{xz}$	viscous stress component on the micro-element surface (Pa)
*f_x_*, *f_y_*, *f_z_*	unit mass force in the *X*, *Y*, *Z* directions (m/s^2^)
$\vec{u}$	velocity of working fluid (m/s)
${\vec{u}}_{p}$	velocity of particles produced by discharging (m/s)
$\rho_{p}{}_{}$	density of the processed products (kg/m^3^)
*μ*	dynamic viscosity of working fluid (Pa·s)
*ρ*	fluid density (kg/m^3^)
*d_p_*	diameter of particles produced by discharging (m)
*Re*	Reynolds number
*C_D_*	drag coefficient
$\vec{g}$	gravitational acceleration (m/s^2^)
*F*	additional forces (N)

## References

[1] Bunker R.S. (2005). A review of shaped hole turbine film-cooling technology. J. Heat Transf..

[2] Bilgi D.S., Jain V.K., Shekhar R., Mehrotra S. (2004). Electrochemical deep hole drilling in super alloy for turbine application. J. Mater. Process. Technol..

[3] Singh H. (2012). Experimental study of distribution of energy during EDM process for utilization in thermal models. Int. J. Heat Mass Transf..

[4] Bamberg E., Heamawatanachai S. (2009). Orbital electrode actuation to improve efficiency of drilling micro-holes by micro-EDM. J. Mater. Process. Technol..

[5] Abbas N.M. (2007). A review on current research trends in electrical discharge machining (EDM). Int. J. Mach. Tools Manuf..

[6] Goodlet A., Koshy P. (2015). Real-time evaluation of gap flushing in electrical discharge machining. CIRP Ann..

[7] Kumagai S., Sato N., Takeda K. (2006). Combination of capacitance and conductive working fluid to speed up the fabrication of a narrow, deep hole in electrical discharge machining using a dielectric-encased wire electrode. Int. J. Mach. Tools Manuf..

[8] Chen S.L., Lin M.H., Huang G.X., Wang C.C. (2014). Research of the recast layer on implant surface modified by micro-current electrical discharge machining using deionized water mixed with titanium powder as dielectric solvent. Appl. Surf. Sci..

[9] Wong Y.S., Lim L.C., Lee L.C. (1995). Effects of flushing on electro-discharge machined surfaces. J. Mater. Process. Technol..

[10] Liu Y., Li M., Niu J., Lu S., Jiang Y. (2019). Fabrication of Taper Free Micro-Holes Utilizing a Combined Rotating Helical Electrode and Short Voltage Pulse by ECM. Micromachines.

[11] Wang W., Zhu D., Qu N., Huang S., Fang X. (2010). Electrochemical drilling with vacuum extraction of electrolyte. J. Mater. Process. Technol..

[12] Yang C.K., Wu K.L., Hung J.C., Lee S.M., Lin L.C., Yan B.H. (2011). Enhancement of ECDM efficiency and accuracy by spherical tool electrode. Int. J. Mach. Tools Manuf..

[13] Hung J.C., Lin J.K., Yan B.H., Liu H.S., Ho P.H. (2006). Using a helical micro-tool in micro-EDM combined with ultrasonic vibration for micro-hole machining. J. Micromech. Microeng..

[14] Nastasi R., Koshy P. (2014). Analysis and performance of slotted tools in electrical discharge drilling. CIRP Ann..

[15] Plaza S., Sanchez J.A., Perez E., Gil R., Izquierdo B., Ortega N., Pombo I. (2014). Experimental study on micro EDM-drilling of Ti6Al4V using helical electrode. Precis. Eng..

[16] Hung J., Liu H., Chang Y., Hung K., Liu S. (2013). Development of helical electrode insulation layer for electrochemical microdrilling. Procedia CIRP.

[17] Fang X.L., Zhang P.F., Zeng Y.B., Qu N.S., Zhu D. (2016). Enhancement of performance of wire electrochemical micromachining using a rotary helical electrode. J. Mater. Process. Technol..

[18] Tang J.P. (2016). ANSYS 16.0.

[19] Morsi S.A., Alexander A.J. (1972). An investigation of particle trajectories in two-phase flow systems. J. Fluid Mech..

